# Mapping the Digital One Health/One Digital Health paradigm: A bibliometric and foresight-based analysis of emerging trends and future directions

**DOI:** 10.1177/20552076261433841

**Published:** 2026-03-19

**Authors:** Jude Kong, Nicola Luigi Bragazzi

**Affiliations:** 1 7938 Africa-Canada Artificial Intelligence and Data Innovation Consortium (ACADIC), Toronto, ON, Canada; 2 Global South Artificial Intelligence for Pandemic and Epidemic Preparedness and Response Network (AI4PEP); 3 Artificial Intelligence & Mathematical Modeling Lab (AIMM Lab), Dalla Lana School of Public Health, University of Toronto, Toronto, ON, Canada; 4 Department of Mathematics, University of Toronto, Toronto, ON, Canada; 5 Department of Computer Science and Information Technology, Faculty of Natural and Applied Sciences, 9379 Sol Plaatje University, Kimberley, South Africa

**Keywords:** One Health, Digital Health, Digital One Health, bibliometric analysis

## Abstract

**Background:**

The One Health (OH) framework, emphasizing the interdependence of human, animal, and environmental health, has expanded with the rise of Digital Health (DH), which applies technologies like artificial intelligence (AI), mobile health, and genomics to improve outcomes. Their intersection, termed Digital One Health (DOH) or One Digital Health (ODH), reflects a major shift in how global health threats are addressed.

**Objectives:**

To map and interpret the thematic structure, evolution, and future frontiers of OH, DH, and their convergence in DOH/ODH through bibliometric and foresight analysis.

**Methods:**

A comprehensive PubMed/MEDLINE search retrieved 18,383 OH articles, 23,281 DH articles, and 290 integrating both. VOSviewer was used to build keyword co-occurrence networks and visualize cluster evolution. Manual thematic labeling and a foresight typology classified clusters as structural drivers, emerging issues, or weak signals.

**Results:**

OH research formed six clusters, including human/veterinary health systems, ecological modeling, and anti-microbial resistance (AMR) governance. DH clustered into seven themes such as digital decision-support, precision diagnostics, and equity-centered interventions. DOH/ODH showed seven integrated clusters (e.g. AI in pandemic response, zoonotic surveillance, and microbial genomics). Over time, a shift toward convergence, prediction, and integration was observed. Foresight mapping identified structural drivers (e.g. digital infrastructure, AMR surveillance), emerging issues (e.g. AI-driven epidemic intelligence, mobile mental health), and weak signals (e.g. occupational biosafety, pathogen phylogenetics, and infodemic management).

**Conclusions:**

DOH/ODH is crystallizing as a cohesive, interdisciplinary paradigm. Advancing ethical governance, equity, and participatory innovation is essential for realizing its global potential.

## Introduction

Pioneered by the American veterinary surgeon and epidemiologist Calvin Schwabe in the mid-20^th^ century,^1^ but formalized only later in the 2000s through coordinated efforts by international organizations,^2^ the “One Health” (OH) framework, centered on the interdependence of human, animal, and environmental health, has emerged in the last decades as a critical paradigm for addressing complex global public health challenges, particularly those situated at the interfaces of zoonotic disease transmission, anti-microbial resistance (AMR), climate change, and food security.^3–5^

With the rise of digital technologies and Digital Health (DH),^6^ the OH approach is undergoing a profound transformation. Innovations such as electronic (eHealth) and mobile health (mHealth), telemedicine, digital surveillance systems, artificial intelligence (AI), and genomic sequencing are not only reshaping how health threats are detected and managed, but also redefining the epistemic foundations of interdisciplinary research and governance in this domain.^7^ The convergence of OH and digital innovation, referred to as “Digital One Health” (DOH) by some scholars,^8,9^ or “One Digital Health” (ODH) by others,^10,11^ represents an emergent research frontier with the potential to enhance early warning capabilities, enable precision public health, and foster cross-sectoral collaboration through real-time data integration and advanced analytics.^7^

Despite increasing policy attention and scientific investment, the conceptual foundations and thematic boundaries of DOH and ODH remain insufficiently defined. Although both frameworks aim to integrate digital technologies into health systems, they diverge in scope and strategic orientation. DOH seeks to strengthen existing OH models by applying targeted digital tools, such as AI, biosensors, and data analytics, to improve surveillance, diagnostics, and intersectoral coordination.^8,9^ In contrast, ODH envisions a more transformative, systems-level redesign of health ecosystems, emphasizing data interoperability and integrated governance across human, animal, and environmental domains.^10,11^ Yet scholarly engagement with these paradigms remains fragmented, often confined within disciplinary silos, and the accelerating pace of technological advancement continues to outpace efforts to map the field's intellectual structure. This underscores the urgent need to clarify the evolving research landscape, delineate its core thematic domains, and illuminate the patterns of interconnection and knowledge production that shape the emerging DOH/ODH ecosystem.

To address this gap, we conducted a comprehensive bibliometric analysis of peer-reviewed literature indexed in a major scholarly electronic biomedical database, referencing both OH and digital innovation. By applying co-occurrence mapping, cluster analysis, and temporal trend analysis, we aimed to conceptualize the thematic architecture of DOH/ODH, trace its epistemological evolution, and identify the dominant and emerging domains of inquiry. Through this analysis, we offer not only a descriptive cartography of the field but also critical insights into its implications for research, policy, and practice. This study is anticipated to contribute to the strategic consolidation of DOH/ODH as an operationalizable framework for 21st-century global public health.

## Materials and methods

This study employed a bibliometric analysis approach to examine the conceptual structure and temporal evolution of research at the intersection of OH and DH. The analysis was conducted in accordance with standard bibliometric methodologies, integrating quantitative mapping with qualitative thematic interpretation,^12,13^ and its reporting was carried out in accordance with established guidelines for bibliometric and scientometric analyses (the “BIBLIO framework”),^14^ ensuring transparency in database selection, search strategy, data extraction, network construction, clustering procedures, and interpretative steps (Supplemental Material).

## Data source and search strategy

A comprehensive search was performed in the PubMed/MEDLINE database to retrieve peer-reviewed publications containing the keywords OH and digital in either the title, abstract, or keyword fields. No language restrictions were applied. The search included articles published from inception up to 9 May 2025 to ensure the inclusion of the most recent contributions. All retrieved records were exported in the PubMed format and subsequently processed for bibliometric analysis.

### Data analysis

The processed bibliometric data were analyzed using VOSviewer (version 1.6.20),^15^ an open-source, widely used tool for constructing and visualizing bibliometric networks. Keyword co-occurrence analysis was conducted combining author-supplied keywords, Medical Subject Headings (MeSH) terms, and terms extracted from titles and abstracts. A minimum occurrence threshold of at least five instances was applied to filter out infrequent terms and enhance interpretability. The full counting method was selected to ensure that all occurrences of keywords were equally weighted.

Using VOSviewer's mapping algorithm based on the association strength normalization method, a co-occurrence network of terms was generated. Terms were grouped into clusters through modularity-based community detection, with each cluster representing a distinct thematic domain. Node size in the visualization corresponds to the frequency of occurrence, while edge thickness reflects the strength of co-occurrence between terms. Colors were used to indicate cluster membership, and spatial positioning represents the degree of conceptual proximity.

To explore the diachronic evolution of thematic trends, a temporal overlay visualization was generated in VOSviewer. Publication years associated with individual keywords were used to calculate the average publication year for each term, allowing for the identification of early, mid-stage, and recently emerging topics. This enabled a temporal gradient to be superimposed on the network map, providing insight into the maturation and shifting focus of the field.

### Interpretative strategy

Following quantitative analysis, each cluster was manually interpreted and labeled based on the dominant keywords and thematic coherence of constituent terms. Special attention was given to cross-cluster linkages, semantic hubs, and peripheral subfields to uncover latent structures and interdisciplinary linkages. These results were then synthesized to construct a conceptual model of DOH/ODH, highlighting both the established research cores and novel frontiers of inquiry.

### Foresight exercise

Finally, to map and interpret the convergence of OH and DH through a futures-oriented lens, we combined bibliometric synthesis with a foresight-driven classification framework. Specifically, we utilized a three-tiered typology widely employed in futures studies, which distinguishes among structural drivers, emerging issues, and weak signals.^16,17^ This framework enables the identification and differentiation of deeply embedded systemic forces, trends gaining traction among stakeholders, and peripheral developments that, despite their current marginality, may hold transformative potential.

The previously identified cluster labels served as semantic anchors for our interpretive process. We systematically analyzed the clusters across the three bibliometric analyses, triangulating conceptual content, visual centrality, cluster size, and temporal dynamics. Clusters that appeared consistently across maps and occupied central positions, reflected well-established domains and were classified as structural drivers, given their institutional embeddedness and historical continuity. Clusters of moderate size that centered on rapidly evolving and shifting paradigms were designated as emerging issues, indicating their increasing relevance and momentum in scholarly and policy discourses. By contrast, clusters characterized by their small scale, peripheral visual positioning, or conceptual novelty were interpreted as weak signals. These were identified as early indicators of potential future disruptions or paradigm shifts at the intersection of OH and DH.

Through this integrative analytic procedure that merged cluster's degree of technological embedding, cross-sectoral relevance, and epistemic maturity, we constructed a foresight-informed conceptual map of the DOH/ODH landscape, revealing not only the dominant structuring forces but also the nascent developments and early signals that may redefine the field's future trajectory.

## Results

The literature search yielded 18,383 publications related to OH and 23,281 pertaining to DH, with a subset of 290 articles addressing both OH and digital innovation, indicating a nascent but growing intersection between the two domains.

### Bibliometric mapping of OH

**Figure 1.**
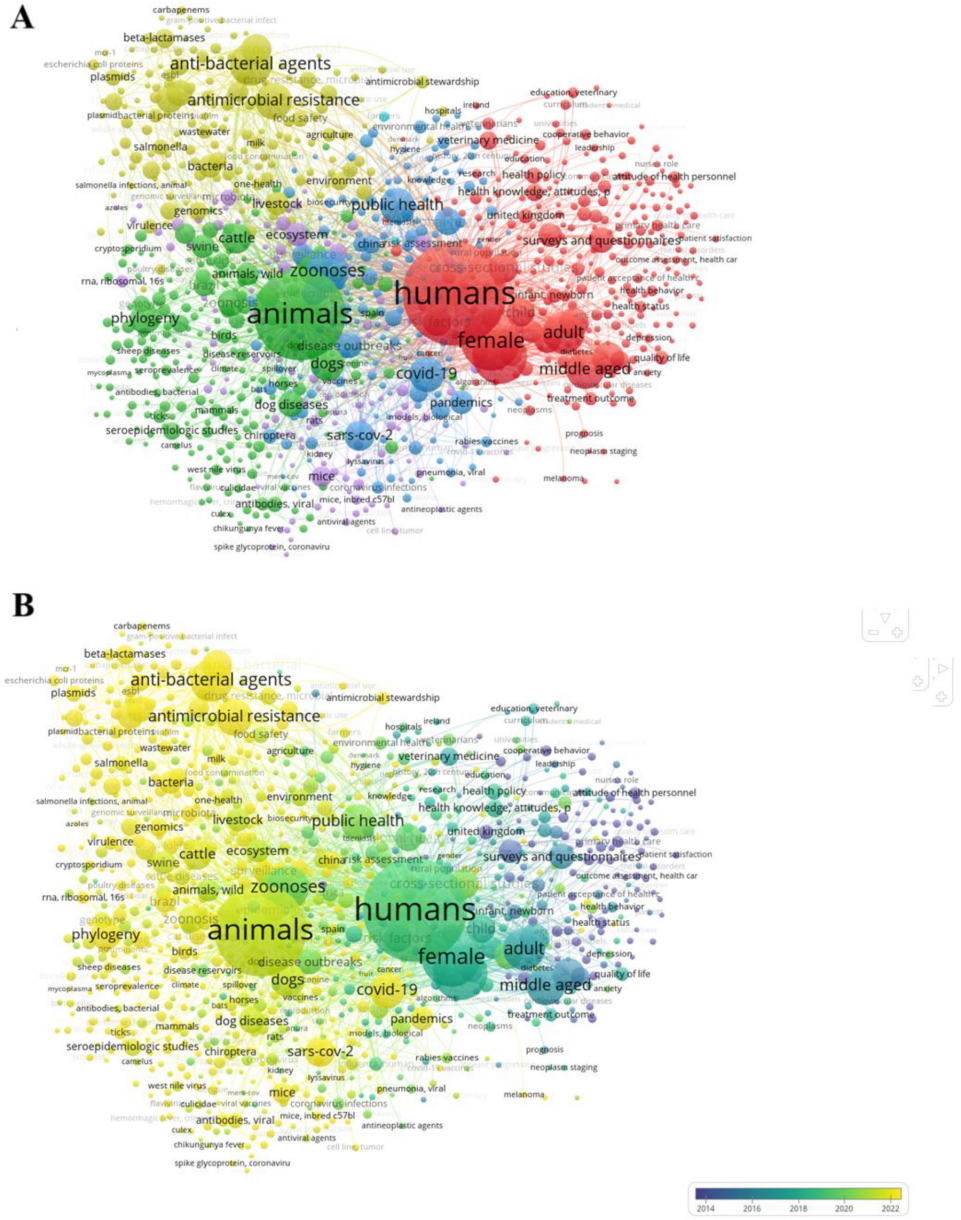
Bibliometric mapping of One Health (a) and its temporal evolution (b).

The bibliometric mapping of OH research revealed a dynamic and stratified, yet convergent landscape, composed of six interdependent thematic domains, reflecting the field's evolution and expansion toward a more integrated, anticipatory, and ecologically grounded paradigm (Figure 1(a)). At the core of this landscape lies Cluster 1 (27.4%), “Human Health Systems in OH,” representing the human-centric nucleus of OH and embodying the legacy of biomedical, epidemiological, and health policy research. Surrounding this nucleus is Cluster 2 (24.0%), “Veterinary-Ecological Systems,” which encompasses the animal–environment *continuum*, consolidating veterinary, ecological, and conservationist traditions. Cluster 3 (17.1%), “Global Health Security and Pandemic Governance,” includes elements of policy, biosecurity, and transnational preparedness, positioning OH as a governance architecture. Interlocking with this policy-focused domain is Cluster 4 (16.4%), “AMR.” This cluster, simultaneously rooted in molecular surveillance and international health governance, exemplifies the operationalization of OH principles through integrated surveillance and stewardship efforts that transcend conventional boundaries between human medicine, veterinary practice, agriculture, and environmental science. Cluster 5 (14.2%), “Precision OH,” embodies OH's mechanistic and computational turn, incorporating molecular biology, toxicogenomics, and systems-level data science. Finally, Cluster 6 (0.3%), although modest in scale, is conceptually significant. “Occupational OH” brings attention to the settings where zoonotic emergence is most acute, underscoring the vulnerabilities of frontline workers.

Temporally (Figure 1(b)), these domains exhibit a discernible evolution with domains such as Cluster 3 expanding markedly in recent years, particularly in response to pandemic disruptions, marking a paradigmatic shift from disciplinary integration to system-level coordination. The earlier focus on clinical and public health themes has been progressively augmented by the rise of ecological, policy-based, and molecular perspectives, transforming OH into a planetary, data-rich framework for predictive and preventive health action.

### Bibliometric mapping of DH

**Figure 2.**
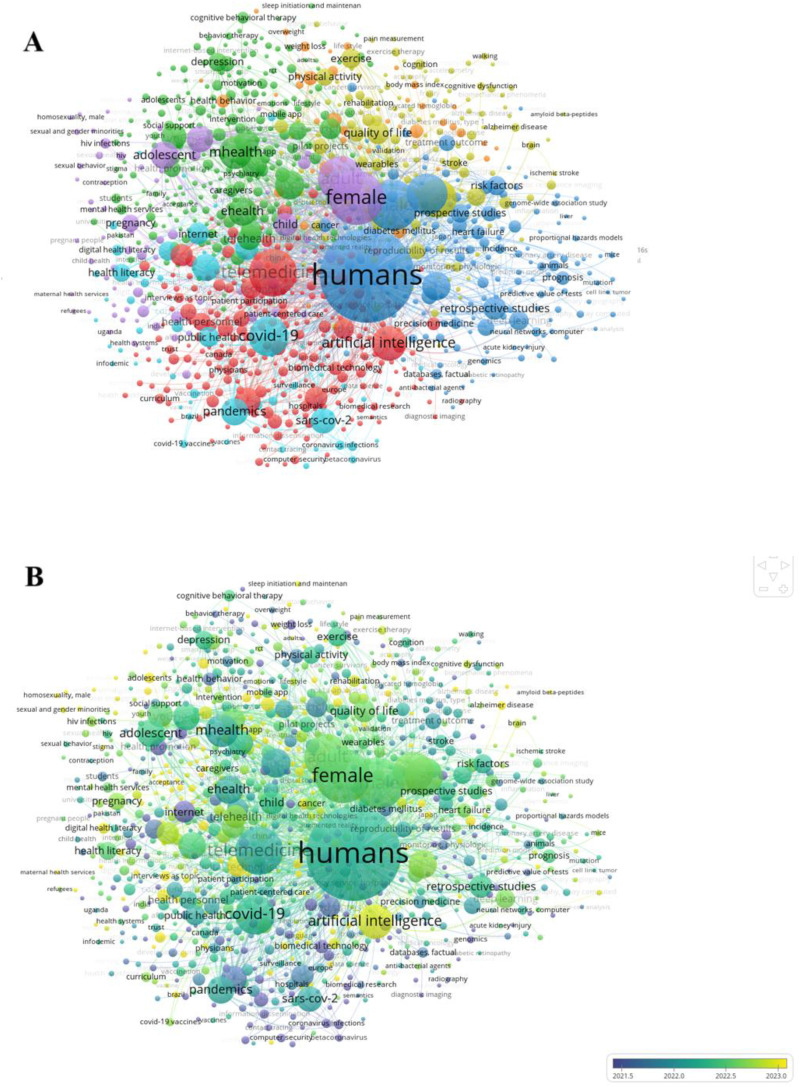
Bibliometric mapping of Digital Health (a) and its temporal evolution (b).

Seven thematically distinct yet conceptually interrelated clusters emerged (Figure 2(a)). Cluster 1 (25.6%), “Decision-Support Systems,” reflects systemic integration of digital infrastructures (AI, clinical decision support, and interoperability) across healthcare systems. These themes represent the technocratic scaffolding underpinning data-driven governance, clinical optimization, and the evolving logic of algorithmic medicine. Cluster 2 (25.0%), “Digital Mental Health and Psychosocial Interventions,” anchors the psychosocial and behavioral dimensions of DH and encapsulates mobile mental health applications, cognitive-behavioral modalities, and user-centered participatory tools. Cluster 3 (17.0%), “Precision DH,” constitutes the molecular and computational core of DH, encompassing high-throughput technologies, machine learning (ML), biomarker discovery, and multiscale risk prediction. Cluster 4 (14.7%), “Aging and Neurodegeneration,” foregrounds the intersection of rehabilitation and sensor-enabled chronic care. Through wearable devices, mobile health systems, and home-based monitoring, it advances a digitally augmented model of aging-in-place and neurocognitive management. Cluster 5 (7.7%), “Global DH for Vulnerable Populations,” reorients attention toward historically underserved populations in low- and middle-income countries. It engages with structural determinants of health, such as gender, poverty, and health system disparities, while exploring mobile technologies and community-based interventions as tools of equity and access. Cluster 6 (5.7%), “DH Communication and Literacy,” traces the diffusion of health information and public opinion across digital platforms, articulating a model of health communication shaped by infodemic management, misinformation surveillance, and social media analytics. Cluster 7 (4.2%), “Lifestyle-Based Digital Interventions,” focuses on disease management, emphasizing lifestyle modification and digital self-monitoring as pillars of noncommunicable disease prevention.

From a temporal perspective (Figure 2(b)), DH has transitioned from siloed, domain-specific models to integrated, digitally enabled, and equity-aware paradigms, with an increasing focus on digital mental health, mHealth, telemedicine, digital literacy, and social media-driven public health discourse.

### Bibliometric mapping of DOH/ODH

**Figure 3.**
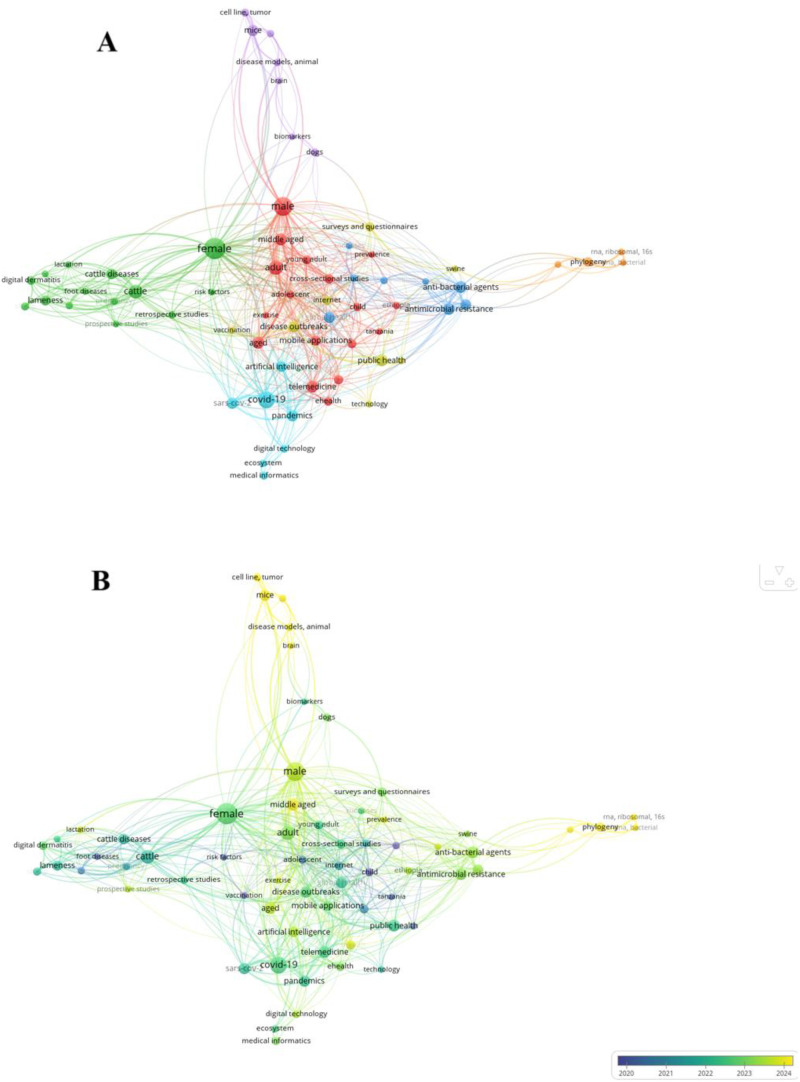
Bibliometric mapping of Digital One Health/One Digital Health (a) and its temporal evolution (b).

Seven distinct yet interconnected thematic clusters emerged within OH-digital landscape (Figure 3(a)). Cluster 1 (26.15%), “Human Health Systems in DOH/ODH,” is characterized by a strong emphasis on the deployment of DH interventions in low-resource settings, disproportionately impacted by zoonotic spillovers, to address disparities in healthcare access and outcomes. Cluster 2 (21.54%), “Veterinary-Ecological Systems in DOH/ODH,” signifies a veterinary epidemiological focus with particular attention to bovine and ovine species. Reproductive and production-related parameters feature prominently, suggesting the integration of digital tools in reproductive monitoring and welfare optimization. Cluster 3 (12.31%), “Global Surveillance and AMR,” bridges public health and microbiological monitoring for coordinated, cross-sectoral responses to transboundary microbial threats. This domain highlights the integration of epidemiological surveillance systems with molecular monitoring tools to track the emergence and dissemination of resistant pathogens across human, animal, and environmental reservoirs. Cluster 4 (12.31%), “Digital Technologies in Epidemic Intelligence and Outbreak Response,” is centered on technology and digital innovation for zoonotic outbreak control, reflecting a digitally enhanced public health infrastructure mobilized for real-time data acquisition and rapid response, particularly at the human-animal interface. It emphasizes how digital infrastructures and informatics platforms are increasingly employed for surveillance, early warning systems, and global coordination in infectious disease control. Cluster 5 (10.77%), “Translational DOH/ODH,” represents a translational research arm of DOH/ODH, wherein animal models are employed to elucidate molecular mechanisms and validate therapeutic targets relevant to both human and animal health, relying on data-intensive approaches, systems biology, and mechanistic modeling. Cluster 6 (10.77%), “AI and Digital Infrastructure in Pandemic Preparedness,” captures the recent acceleration of DH and OH research in response to the COVID-19 pandemic, which served as a catalyst for cross-sectoral integration of digital tools in epidemiology, policy-making, and health communication. Cluster 7 (6.15%), “Microbial Genomics and Molecular Phylogenetics,” is situated at the genomic frontier of the OH paradigm, exemplifying the application of next-generation sequencing and molecular taxonomy in pathogen discovery, evolutionary tracing, and microbial ecology.

From a temporal perspective (Figure 3(b)), OH has progressively transitioned from an initial emphasis on foundational epidemiological and demographic descriptors to a focus on technological augmentation and predictive modeling. This evolution marks a paradigmatic shift from reactive disease control to proactive, digitally mediated governance frameworks, underscoring the emergence and consolidation of DOH/ODH as a planetary surveillance and response infrastructure, one increasingly capable of integrating multi-species health signals, environmental data streams, and behavioral metrics in real time.

### Foresight exercise of DOH/ODH

When interpreting the bibliometric landscape derived from the convergence of OH and DH research through the foresight framework, one of the structural drivers centered on health system strengthening through digital means, particularly in resource-constrained and zoonotically vulnerable regions. This theme, deeply rooted in the OH literature through its focus on human health systems and in DH through digital infrastructure and equity-driven applications, emerged as the principal structural axis in the OH/DH convergence. A second structural driver was the domain uniting veterinary and ecological systems with digital surveillance tools, an area with strong representation in both OH (as ecological and conservationist traditions) and DH (*via* sensor-based monitoring and remote diagnostics). The third robust structural domain bridged cross-sectoral AMR with digital epidemiological platforms. Although AMR has long been a mainstay of OH, its digital operationalization, *via* genomic tools, dashboards, and policy-linked informatics, appeared as a consolidating force in the OH/DH subset.

One of the emerging issues encompassed the growing use of AI and ML in epidemic intelligence, surveillance, and response. While this theme was prominent in the DH map under algorithmic medicine and decision-support systems, it appeared in the OH landscape primarily as a reactive, post-COVID expansion. Another emergent domain involved translational and mechanistic research leveraging animal models and data-intensive methods to address shared human–animal health problems. This cluster drew from OH's interest in comparative pathobiology and DH's emphasis on precision technologies, forming a novel bridge in the converged space. Additionally, mobile mental health and psychosocial interventions, though primarily embedded in DH, gained relevance for OH during the COVID-19 pandemic and related zoonotic crises, signaling a cross-cutting behavioral dimension entering the convergence space.

Weak signals included the use of genomic and phylogenetic methods for pathogen discovery and ecological tracing, which, despite their methodological sophistication, remained on the periphery of both the DH and OH discourses and were still loosely articulated in the converged map. Another weak signal was the emergence of occupational health concerns, particularly related to biosafety and zoonotic exposure among frontline workers. While present in OH under zoonotic emergence contexts and in DH through telemedicine and labor monitoring technologies, this theme had not yet coalesced into a fully institutionalized or strategic focus within the OH/DH literature. A third weak signal involved health communication, infodemic management, and DH literacy. Despite strong attention in the DH corpus, these issues remained under-integrated in the OH field and appeared in the OH/DH map only as a loosely connected periphery cluster, pointing to a latent but underexplored sociotechnical dimension of convergence.

Beyond its descriptive value, the foresight analysis offers actionable insights for policy and strategic planning. Structural drivers identified in this study, such as digital infrastructure for surveillance, cross-sectoral AMR monitoring, and integrated data systems, point to immediate policy priorities, including sustained investment in interoperable health information architectures and the institutionalization of cross-ministerial coordination mechanisms spanning human, animal, and environmental health. Emerging issues, particularly AI-driven epidemic intelligence and mobile health interventions, suggest near-term scenarios in which regulatory frameworks, ethical oversight, and workforce capacity-building will become critical determinants of successful implementation. In contrast, weak signals (such as occupational biosafety, microbial phylogenetics, and infodemic management) outline longer-term or disruptive scenarios that may require anticipatory governance, adaptive regulation, and early-stage pilot programs to prevent future systemic vulnerabilities. Framing these domains within a foresight lens thus enables the translation of bibliometric patterns into policy-relevant scenarios, distinguishing between areas requiring immediate action, strategic experimentation, or long-term preparedness, and enhancing the practical relevance of the DOH/ODH paradigm for decision-makers.

## Discussion

This bibliometric and network-based study reaffirms the growing consolidation of DOH/ODH as an emergent epistemic and operational framework. Through systematic co-occurrence mapping and diachronic analysis, the investigation elucidates how the hybridization of OH with digital technologies has shifted the field's center of gravity, from traditional surveillance and siloed biomedical paradigms to a digitally augmented, integrative, and anticipatory model of planetary health governance. The convergence between human, animal, and environmental health is increasingly mediated by digital infrastructures, AI, and computational tools, transforming both the nature of data and the scope of intervention. The thematic clusters identified in the DOH/ODH literature reflect a multi-scalar architecture. Domains such as “Global Surveillance and AMR” and “Digital Technologies in Epidemic Intelligence” showcase the functional synthesis of digital and microbiological monitoring, particularly relevant in light of recent pandemic experiences. Meanwhile, clusters such as “Microbial Genomics and Molecular Phylogenetics” and “Translational DOH/ODH” reveal a molecular substratum underpinning this synthesis, with high-throughput sequencing and animal models serving as core components. Notably, the presence of clusters like “Veterinary-Ecological Systems in DOH/ODH” and “Human Health Systems in DOH/ODH” illustrates that traditional pillars of OH remain intact but have been digitally reconfigured. Temporal trends confirm a diachronic evolution in which earlier biomedical descriptors have given way to algorithmic, data-driven, and computational terms, marking a decisive shift toward digitally mediated forms of knowledge production and governance.

Despite these advances, several gaps remain. First, while the DOH/ODH field increasingly integrates AI/ML and genomic surveillance, it remains underdeveloped in terms of ethical, legal, and social implications (ELSI). The normative frameworks that should accompany the deployment of such technologies are, indeed, poorly represented in the current scholarly literature. However, although ELSI remain underrepresented in the current DOH/ODH literature, their relevance is increasingly evident as digital infrastructures mediate cross-species surveillance and decision-making. Key ethical challenges include data governance and sovereignty across human, animal, and environmental domains, particularly in low- and middle-income settings where asymmetries in technological capacity may exacerbate existing inequities. The use of AI in epidemic intelligence and zoonotic surveillance raises concerns related to algorithmic transparency, bias, accountability, and the delegation of decision-making authority in public health emergencies. Furthermore, the large-scale integration of genomic, mobility, and behavioral data introduces tensions between population-level benefits and individual or community-level rights, including privacy, consent, and trust. Social implications are also salient, as digitally mediated OH interventions may unintentionally marginalize local knowledge systems or reinforce technocratic governance if not accompanied by participatory and context-sensitive implementation strategies. Addressing these issues requires the development of explicit ethical frameworks and governance models capable of balancing innovation, equity, and accountability within the DOH/ODH paradigm.

Second, there is a paucity of studies focusing on participatory governance models or community-driven digital innovation, particularly in low-resource settings, where DOH/ODH could play a transformative role. Third, the conceptual boundaries between clusters, especially those involving “Translational DOH/ODH” and “AI in Pandemic Preparedness,” remain fluid, suggesting the need for theoretical refinement and clearer operational definitions. Lastly, planetary-scale DOH/ODH frameworks capable of integrating climate data, biodiversity metrics, and behavioral indicators remain more aspirational than empirically realized.

This present study adds to the existing literature by offering a more expansive and theoretically grounded analysis of the DOH/ODH paradigm, extending beyond previous contributions. Miao et al.^18^ adopted a descriptive bibliometric approach grounded in the Scopus database to map the trajectory of OH research from 2003 to 2021, identifying four major research areas of OH: namely, (i) zoonotic diseases, (ii) vector-borne infections, (iii) AMR, and (iv) food safety. Furthermore, the analysis highlighted an exponential increase in OH-related publications, particularly in the wake of pandemics such as Zika and COVID-19, and emphasized the dominance of the natural sciences, with limited engagement from social sciences or governance frameworks. Research gaps could be pinpointed: most notably, the marginalization of environmental health and insufficient interdisciplinarity. Hu et al.^19^ assessed the evolving intellectual structure of DH, which has shifted from early focuses on internet-based health communication and electronic health records to current emphases on AI, mental health, and patient empowerment. Scott et al.^7^ conducted the only existing bibliometric study specifically focused on DOH/ODH. However, they primarily emphasized biomedical informatics applications and MeSH-driven thematic clustering.

By integrating a significantly broader dataset and deploying both modularity-based network detection and temporal overlay visualization, this study delineates a more nuanced, multiscalar architecture of DOH/ODH that spans molecular, clinical, ecological, and governance domains. Furthermore, unlike earlier analyses that remain largely descriptive, this investigation introduces critical reflections on ethical governance, equity, and the epistemological boundaries of the field, thereby transforming DOH/ODH from a conceptual extension of OH into a consolidated interdisciplinary knowledge system.

Moreover, the main finding from the bibliometric and diachronic evidence presented in this study is the consolidation of DOH as the prevailing paradigm emerging from the convergence of OH and digital innovation. While both DOH and ODH seek to integrate digital technologies into health frameworks, their conceptual orientations diverge: DOH emphasizes the operational enhancement of existing OH models through targeted deployment of digital tools, whereas ODH envisions a more transformative, systems-level reconfiguration of health governance across species and ecological domains. The thematic clusters identified in the DOH/ODH corpus, particularly those centered on zoonotic surveillance, pandemic intelligence, AMR, and translational informatics, reflect a pragmatic, tool-based integration of digital capacities within established OH infrastructures, aligning more closely with the DOH framework. The shift toward ODH remains more theoretical and less empirically substantiated.

To enhance conceptual clarity and better interpret this observed asymmetry, it is useful to explicitly articulate the distinction between DOH and ODH as analytically distinct yet related paradigms. As previously stated, DOH can be understood as an operational extension of the traditional OH framework, in which targeted digital tools, such as AI-driven surveillance, genomic sequencing, decision-support systems, and interoperable data platforms, are embedded within existing human, animal, and environmental health structures to strengthen surveillance, preparedness, and response. In this sense, DOH is predominantly problem-driven and application-oriented, which helps explain its coherent and empirically grounded representation within the bibliometric clusters identified in this study, particularly those related to zoonotic surveillance, AMR, epidemic intelligence, and pandemic preparedness. By contrast, ODH represents a more normative and systems-level vision of health governance, proposing a digitally native health ecosystem in which multispecies and environmental data streams are integrated from the outset through shared infrastructures, harmonized standards, and coordinated governance mechanisms. ODH places stronger emphasis on ethical governance, data sovereignty, interoperability, and participatory design, implying a transformative reconfiguration of health systems rather than the incremental digital enhancement of existing ones. As a consequence, ODH currently remains more aspirational and conceptually diffuse, with fewer empirically consolidated applications, a pattern that is reflected in its more peripheral and fragmented bibliometric footprint. Rather than representing competing frameworks, DOH and ODH can therefore be interpreted as positions along a *continuum* of digital integration within OH, with DOH capturing the present operational maturity of the field and ODH delineating a future-oriented trajectory that has yet to be fully instantiated. This distinction not only clarifies the conceptual landscape but also provides a coherent explanatory lens for the empirical patterns observed in the present analysis, while paving the way for future research aimed at operationalizing the intersection between OH and DH and empirically testing its added value across integrated human, animal, and environmental health systems.

### Strengths and limitations

The strengths of this study lie in its rigorous methodological integration of bibliometric mapping and temporal overlay analysis, offering both structural and evolutionary insights into the DOH/ODH research landscape. Its use of co-occurrence networks and modularity-based clustering facilitates a robust conceptual segmentation of the field while preserving inter-cluster connectivity. Moreover, by manually interpreting clusters with attention to cross-disciplinary linkages, the study avoids reductive keyword-counting and instead provides a nuanced reading of the field's intellectual evolution. However, limitations must be acknowledged. The reliance on PubMed/MEDLINE as the sole data source, although ensuring high-quality biomedical coverage and standardized indexing, may exclude relevant publications indexed in interdisciplinary or regional databases, potentially introducing a bias. Furthermore, the keyword-based approach may miss latent themes that are not explicitly lexicalized. Lastly, while the temporal overlay provides valuable insights into diachronic dynamics, it cannot fully capture the contextual forces (e.g. pandemics, policy shifts) that may drive sudden accelerations or redirections in research focus.

Future research could extend the present work by adopting a multi-database retrieval strategy that integrates biomedical, interdisciplinary, and regional indexing platforms (e.g. Engineering Village (Ei Compendex), Scopus, or ISI/Web of Science), thereby enabling a more comprehensive representation of the sociotechnical, policy, and systems-science dimensions of DOH/ODH. Methodologically, hybrid bibliometric approaches that combine keyword-based mapping with citation-, co-citation-, or full-text semantic analyses may help uncover latent or conceptually adjacent themes that are not explicitly lexicalized. Longitudinal designs incorporating policy timelines, major outbreak events, and technological inflection points could further contextualize observed diachronic shifts and better disentangle endogenous scientific evolution from exogenous drivers. Finally, future studies should move beyond descriptive cartography toward evaluative and translational analyses, assessing how identified thematic clusters are operationalized in practice, embedded within governance frameworks, and translated into measurable health, equity, and resilience outcomes across human, animal, and environmental systems.

## Conclusions

Our analyses showed that DOH/ODH is a rapidly maturing field characterized by increasing interdisciplinarity, methodological sophistication, and digital convergence. The OH-digital research nexus has transformed from a conceptual framework into a dynamic, multi-scalar research ecosystem that spans the molecular to the planetary, affirming its critical role in addressing 21st-century health challenges. However, gaps remain to truly become a unified knowledge system, integrating biology, ecology, informatics, governance, and equity, to model and manage the interdependencies that define health in the Anthropocene. The current research landscape, as evidenced by bibliometric clustering and publication trajectories, privileges DOH as an operationally mature and epistemologically grounded paradigm, while ODH remains an emergent, albeit underdefined, conceptual extension. Future efforts to advance ODH should move beyond abstract theorization and offer clear, practical frameworks for implementation. This would entail developing demonstrable use cases, scalable interventions, and governance models.

## Supplemental Material

sj-docx-1-dhj-10.1177_20552076261433841 - Supplemental material for Mapping the Digital One Health/One Digital Health paradigm: A bibliometric and foresight-based analysis of emerging trends and future directions Supplemental material, sj-docx-1-dhj-10.1177_20552076261433841 for Mapping the Digital One Health/One Digital Health paradigm: A bibliometric and foresight-based analysis of emerging trends and future directions by Jude Kong and Nicola Luigi Bragazzi in DIGITAL HEALTH
